# Second primary cancer in survivors following concurrent chemoradiation for locally advanced non-small-cell lung cancer

**DOI:** 10.1038/sj.bjc.6603422

**Published:** 2006-10-10

**Authors:** N Takigawa, K Kiura, Y Segawa, Y Watanabe, H Kamei, T Moritaka, T Shibayama, H Ueoka, K Gemba, T Yonei, M Tabata, T Shinkai, S Hiraki, M Takemoto, S Kanazawa, K Matsuo, M Tanimoto

**Affiliations:** 1 Department of Hematology, Oncology, and Respiratory Medicine, Okayama University Graduate School of Medicine, Dentistry, and Pharmaceutical Sciences & Okayama University Hospital, Okayama 700-8558, Japan; 2 Department of Medicine and Thoracic Oncology, National Hospital Organization, Shikoku Cancer Center, Matsuyama 791-0288, Japan; 3 Department of Internal Medicine, Okayama Red Cross Hospital, Okayama 700-8607, Japan; 4 Department of Internal Medicine, Sumitomo Besshi General Hospital, Niihama 792-8543, Japan; 5 Department of Pulmonary Medicine, Ehime Prefectural Central Hospital, Matsuyama 790-0024, Japan; 6 Department of Pulmonary Medicine, National Hospital Organization, Minami-Okayama Medical Center, Tsukubo 701-0304, Japan; 7 Department of Medicine, National Hospital Organization, Sanyo Hospital, Ube 755-0241, Japan; 8 Department of Pulmonary Medicine, Okayama Rosai Hospital, Okayama 702-8055, Japan; 9 Department of Pulmonary Medicine, Okayama Medical Center, Okayama 701-1192, Japan; 10 Department of Radiology, Okayama University Graduate School of Medicine, Dentistry, and Pharmaceutical Sciences & Okayama University Hospital, Okayama 700-8558, Japan; 11 Department of Epidemiology and Prevention, Aichi Cancer Center Research Institute, Nagoya 494-8681 Japan

**Keywords:** non-small-cell lung cancer, chemotherapy, radiotherapy, second primary cancer

## Abstract

Long-term cancer survivors risk development of second primary cancers (SPC). Vigilant follow-up may be required. We report outcomes of 92 patients who underwent chemoradiation for unresectable stage III non-small-cell lung cancer, with a median follow-up of 8.9 years. The incidence of SPC was 2.4 per 100 patient-years (95% confidence interval: 1.0–4.9).

Second primary cancer (SPC) and secondary leukaemia have been recognised in survivors of non-metastatic non-small-cell lung cancer (NSCLC) (Duchateau and Stokkel, 2005). In one study in which, 547 patients were treated with chemoradiation (CRT) using a variety of chemotherapeutic regimens, radiation (RT) doses, and schedules (Kawaguchi *et al*, 2006). Sixty-two patients were disease free more than 3 years, but nine of them developed SPC with an incidence estimated at 2.9 per 100 patient-years. Furthermore, the causes of death in long-term survivors of advanced NSCLC have not been fully described. More data are required to understand the risk of SPC and to establish guidelines for follow-up after combined modality treatment.

We previously reported the 2- or 3-year survival outcomes of two concurrent CRT regimens in surgically unresectable stage III NSCLC (Segawa *et al*, 2000; Kiura *et al*, 2003). Here we report long-term results on cause of death and analysis of SPC in these two trials with a minimum follow-up period of 5 years.

## PATIENTS AND METHODS

### Patients

Between January 1994 and December 1999, two phase II concurrent CRT studies for locally advanced unresectable stage IIIA and IIIB NSCLC in a total of 92 patients were conducted. Briefly, patients had histologically or cytologically confirmed NSCLC, previously untreated disease, measurable lesions, age ⩽75 years, and no history of malignancy within 5 years of enrollment. Any patients who had previously undergone chemotherapy or radiotherapy were excluded. Institutional review boards approved study protocols, and patients provided written informed consent. Treatment in one study consisted of three cycles of 5-fluorouracil (500 mg/m^2^, days 1–5) and cisplatin (FP-RT) (20 mg/m^2^, days 1–5), every 4 weeks, and concurrent hyperfractionated RT (1.25 Gy twice daily, total dose: 62.5–70 Gy) (FP-RT). In the second study, treatment consisted of combination docetaxel 40 mg/m^2^ and cisplatin (DP-RT) 40 mg/m^2^, days 1, 8, 29, and 36 with concurrent RT (2 Gy daily, total 60 Gy) (DP-RT). For the first 2 years following therapy, patients were monitored monthly with chest radiography and every 6 months with computed tomography (CT) (chest and abdomen) and magnetic resonance imaging (brain). After 2 years, chest radiography was performed annually whereas annual brain MRI and semi-annual CT continued. SPC in the lung was defined according to the methods of Martini and Melamed (1975). Patients’ status was described according to Eastern Cooperative Oncology Group performance status (PS).

### Statistical analysis

Statistical analyses were performed using the SPSS program version 11.0J (SPSS Inc., Chicago, IL, USA). Survival time was defined as the period from CRT treatment initiation to last follow-up evaluation or death. SPC rate was calculated as the ratio of SPC cases over 100 patient-years of follow-up (Thomas and Rubinstein, 1993; Jeremic *et al*, 2001).

The interval of initial NSCLC to SPC was measured from the initiation of CRT to SPC diagnosis, where the date of first clinical evidence of histologically or cytologically confirmed SPC was used. Survival rates and cumulative risks of developing SPC were calculated using Kaplan and Meier method and differences in survival distribution between two categorised groups were assessed using a log-rank test. *P*-values less than 0.05 in two-tailed analyses were considered significant. Analyses of these data are based on information available as of 1 October 2005. Data for patients still alive at last follow-up or dead without developing SPC were censored when calculating the cumulative risks.

## RESULTS

**Figure 1:**
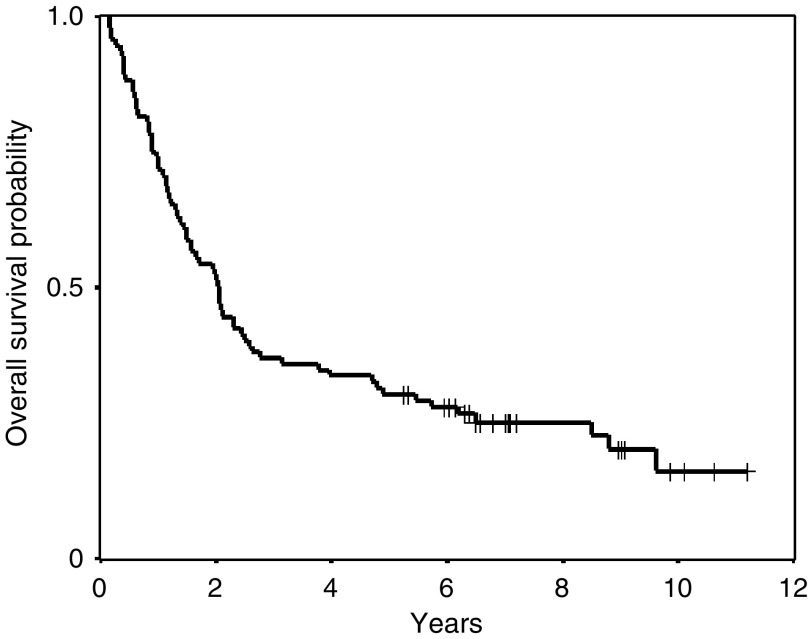
Overall survival curve from the initiation of concurrent CRT.

In the first study, 50 patients were treated with FP-RT and in the second study 42 patients were treated with DP-RT. Among these 92 patients were 81 men and 11 women. At treatment initiation, 21 patients had stage IIIA and 71 patients had stage IIIB disease. The median age for all patients was 65 years (range; 29–75 years). Performance status was 0 (*n*=35), one (*n*=54) and two (*n*=3). With a median follow-up time of 8.9 years (range, 5.2–11.4 years), the observed 5-year survival rate was 30%, with 28 of 92 patients surviving more than 5 years. Median survival time was 2.0 years (95% confidence interval (CI): 1.6–2.5 years) (Figure 1). In the FP-RT study the 5-year survival rate was 30% and in the DP-RT study it was 31%. The median survival times were 1.6 years (95% CI: 0.91–2.25 years) and 2.1 years (95% CI: 0.82–2.5 years), respectively.

Among the 92 patients, two did not complete the originally planned treatment. One had a treatment-related death and another developed cerebral infarction; they were therefore not included in this analysis. Of the remaining 90 patients, 61 (68%) eventually developed disease progression. The most common site of initial failure was within the site of original disease (local progression only, *n*=28; local plus distant progression, *n*=8; distant progression only *n*=24; unspecified, *n*=1). Brain metastases were observed in eight patients, and for six it was the only site of relapse. Among the 28 patients who survived more than 5 years, four died owing to progression of primary NSCLC, three died owing to SPC, two were lost to follow-up, and 19 were alive. Four of the 19 long-term survivors developed SPC (two NSCLC, one small-cell lung cancer, and one oesophageal cancer). Thus, from 90 patients initially included in the two studies a total of seven (7.8%) developed SPC (two NSCLC, one small-cell lung cancer, two oesophageal cancer, and two biliary tract cancer) (Table 1). No patients developed leukaemia or myelodysplastic syndrome. The two patients with second NSCLC (both stage IA) underwent surgery, whereas the patient with small-cell lung cancer (limited disease) received chemotherapy. All were alive at the time of data cutoff. In the two patients with oesophageal cancer, one was alive and the other had died at the time of data cutoff. The former had cancer detected by routine endoscopy, in the absence of symptoms; the latter was diagnosed with advanced disease after developing dysphagia. The patient with gall bladder cancer reported abdominal pain, but despite surgery with curative intent, died of SPC. The patient with bile duct cancer presented with jaundice, received palliative chemotherapy, and died of SPC. All patients who developed SPC had a history of smoking, but only one continued after the diagnosis of primary NSCLC was made. Only one SPC arose in the RT treatment of initial NSCLC.

**Figure 2:**
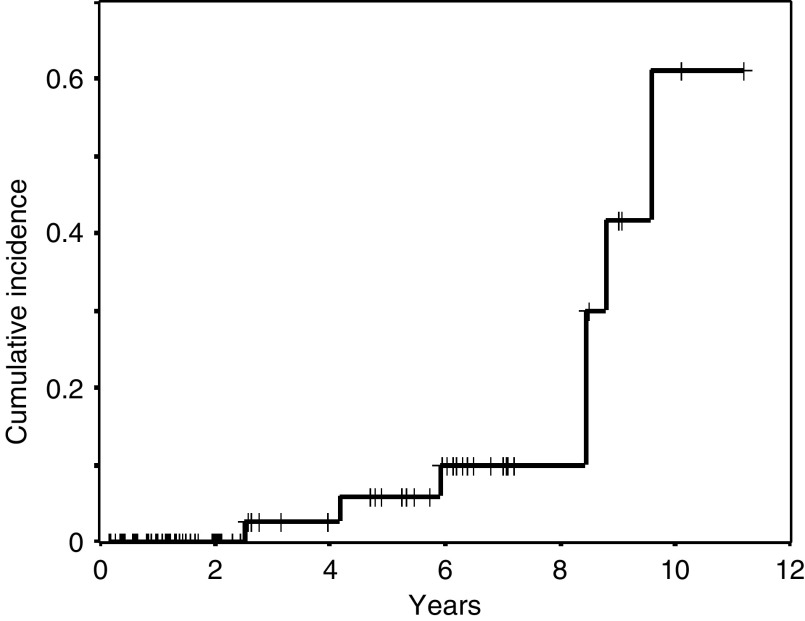
Cumulative incidence of SPC from the initiation of concurrent CRT.

The observed incidence rate of SPC was 2.4 per 100 patient-years (95% CI: 1.0–4.9). Cumulative incidence was 2.7% (standard error s.e. 2.6%) at 3 years, 5.8 % (s.e. 4.0%) at 5 years, 10.0% (s.e. 5.6%) at 8 years, 41.8% (s.e. 15.3%) at 9 years, and 60.8% (s.e. 18.9%) at 10 years (Figure 2). The median time from the beginning of CRT to the diagnosis of SPC was 9.6 years (95% CI: 8.1–11.1 years). All patients with SPC showed no progressive disease of the primary NSCLC.

## DISCUSSION

The analysis of data after a median 8.9 years demonstrates that for patients treated with CRT for unresectable NSCLC in our previous studies, the 5-year-survival rate was 30%. In the patients who underwent rigorous follow-up detection of SPC was assured. Three primary lung cancers were detected during routine follow-up and were curatively treated. Routine follow-up in this study seemed useful to identify early stage lung cancer, however, additional examinations such as ultrasonic abdominal imaging, occult blood stool tests, endoscopy, or measurement of tumour markers may also be beneficial for early detection of alimentary canal cancer in such patients with high-risk of SPC. Further investigation of follow-up methods including cost-benefit analysis is necessary.

The studies using the person-years method to calculate the incidences of SPC showed a range from 1.7 to 4.3 per 100 patient-years (Thomas and Rubinstein, 1993; Ginsberg and Rubinstein, 1995; Martini *et al*, 1995; Jeremic *et al*, 2001; Kawaguchi *et al*, 2006). In the present studies, the rate of SPC was 2.4 per 100 patient-years, in agreement with previous findings. Chemotherapy and RT may be carcinogenic treatments and may have contributed to SPC compared with surgery for early NSCLC (Allan and Travis, 2005). Patients with locally advanced NSCLC still have poor prognosis. Concurrent platin-containing CRT prolongs survival (Aupérin *et al*, 2006), despite the increased risk of SPC in long-term survivors. In this study, it is difficult to speculate on the association of SPC with RT because only one SPC was present in the RT field.

Despite the small sample size in these studies, a significant number of patients treated with concurrent CRT for locally advanced unresectable stage IIIA and IIIB NSCLC survived more than 5 years. Brain metastases in long-term NSCLC survivors have been widely reported and have been the focus of several studies (Carolan *et al*, 2005; Gaspar *et al*, 2005). As we observed that SPC occurs just as frequently, long-term survivors require vigilant follow-up to detect SPC at the earliest possible stage. Studies to find methods to prevent SPC in such patients are warranted.

## Figures and Tables

**Table 1:** Characteristics of seven patients with SPC following chemoradiation

**Type of SPC**	**First to second cancer (years)**	**Smoking (pack-year)**	**Chemo**	**RT field**	**First symptom**	**Treatment of SPC**	**Survival after SPC (years)**
Gall bladder	2.5	100	DP	Outside	Abdominal pain	Curative operation	1.2 (died of SPC)
Oesophagus	4.1	30	FP	Outside	None	Curative chemo RT	7.2 (alive)
Lung (adeno)	5.9	30	DP	Outside	None	Curative operation	1.6 (alive)
Oesophagus	8.3	50	FP	Inside	Dysphagia	Palliation	0.35 (died of SPC)
Lung (adeno)	8.3	68*	FP	Outside	None	Curative operation	1.5 (alive)
Bile duct	8.7	40	FP	Outside	Jaundice	Palliative chemo	0.79 (died of SPC)
Lung (small)	9.5	75	FP	Outside	None	Chemo	1.1 (alive)

Adeno=adenocarcinoma; chemo=chemotherapy; DP=docetaxel and cisplatin; FP=5-fluorouracil and cisplatin; RT=radiation; small=small-cell carcinoma; SPC=second primary cancer;

* The number of 68 was the value at the time of diagnosis of initial NSCLC and this patient had continued smoking as SPC was found.
